# Impulsivity and compulsivity aggregate in alcohol use disorder and explain comorbidity with impulse-control and related disorders

**DOI:** 10.1192/j.eurpsy.2021.1517

**Published:** 2021-08-13

**Authors:** A. Araujo, A.T. Pereira, C. Reis, L. Nascimento, C. Pina, J. Avó, A. Feijão, A. Macedo

**Affiliations:** 1 Department Of Psychological Medicine, Faculty of Medicine, University of Coimbra, Coimbra, Portugal; 2 Psychiatry Department, Centro Hospitalar e Universitário de Coimbra, Coimbra, Portugal; 3 Institute Of Psychological Medicine, Faculty of Medicine, University of Coimbra, Coimbra, Portugal; 4 Faculty Of Medicine, University of Coimbra, Coimbra, Portugal; 5 Dicad, ARS, Centro, Coimbra, Portugal; 6 Institute Of Psychological Medicine, Faculty Of Medicine, University of Coimbra, Coimbra, Portugal

## Abstract

**Introduction:**

The conceptualization of impulsivity and compulsiveness has fluctuated between two different perspectives: they are (1) distinct and orthogonal dimensions, (2) extreme poles of the same dimension/ spectrum. We favor this latter, accepting that these dimensions contribute to the etiopathogenesis of impulsive-compulsive disorders, namely alcohol use disorder/AUD.

**Objectives:**

To analyze: Differences of impulsivity and compulsivity levels between AUD patients vs. participants from the community; prevalence of impulsive-compulsive disorders/ICD in AUD; if impulsivity/compulsivity predict the severity of alcohol use and ICD in AUD.

**Methods:**

32 AUD patients (21% women, mean age 46±10) answered the Portuguese versions of: Alcohol-Use-Disorders-Identification-Test, Questionnaire-for-Impulsive-Compulsive-Disorders-in-Parkinson’s-Disease, Barrat-Impulsiveness-Scale, Obsessive-Compulsive-Inventory and Depression-Anxiety-Stress-Scales; 50 adults from the community (68% women, mean age 29±14) answered the former three. Mann-Whitney-U, Spearman and regression tests were performed using SPSS.

**Results:**

AUD individuals vs. subjects from the community presented higher levels of impulsivity and compulsivity (p<.001). AUD-group: AUDIT median score was 25 (>8 harmful use); 81% reported ICD-symptoms; impulsivity and compulsivity highly correlated (r=.639; p<.001); impulsivity levels explained the presence of certain ICD (gambling, compulsive buying, eating disorders) and depression/anxiety/stress (OR=.152; p<.05); compulsivity levels also explained the occurrence of specific ICD (compulsive buying and other repetitive automatic behaviours) and depression/anxiety/stress (OR=.131 p<.05).

**Conclusions:**

Our results indicate that impulsivity and compulsivity co-occur and contribute to the explanation of AUD, and related comorbidity and psychological distress. This highlights the utility of considering impulsivity and compulsivity when subtyping, stratifying, and treating AUD patients. Finally, we assert that disorders of impulsivity and compulsivity (eg.: AUD and ICD) co-occur.

**Conflict of interest:**

No significant relationships.

